# A radiomics model fusing clinical features to predict microsatellite status preoperatively in colorectal cancer liver metastasis

**DOI:** 10.1186/s12876-023-02922-0

**Published:** 2023-09-12

**Authors:** Xuehu Wang, Ziqi Liu, Xiaoping Yin, Chang Yang, Jushuo Zhang

**Affiliations:** 1 College of Electronic and Information Engineering, Hebei University, Baoding, 071002 China; 2 Research Center of Machine Vision Engineering & Technology of Hebei Province, Baoding, 071002 China; 3 Key Laboratory of Digital Medical Engineering of Hebei Province, Baoding, 071002 China; 4 Affiliated Hospital of Hebei University, Bao Ding, 071000 China

**Keywords:** Radiomics, Logistic regression, Liver metastasis of colorectal cancer, Nomogram, Microsatellite instability

## Abstract

**Purpose:**

To study the combined model of radiomic features and clinical features based on enhanced CT images for noninvasive evaluation of microsatellite instability (MSI) status in colorectal liver metastasis (CRLM) before surgery.

**Methods:**

The study included 104 patients retrospectively and collected CT images of patients. We adjusted the region of interest to increase the number of MSI-H images. Radiomic features were extracted from these CT images. The logistic models of simple clinical features, simple radiomic features, and radiomic features with clinical features were constructed from the original image data and the expanded data, respectively. The six models were evaluated in the validation set. A nomogram was made to conveniently show the probability of the patient having a high MSI (MSI-H).

**Results:**

The model including radiomic features and clinical features in the expanded data worked best in the validation group.

**Conclusion:**

A logistic regression prediction model based on enhanced CT images combining clinical features and radiomic features after increasing the number of MSI-H images can effectively identify patients with CRLM with MSI-H and low-frequency microsatellite instability (MSI-L), and provide effective guidance for clinical immunotherapy of CRLM patients with unknown MSI status.

## Introduction

Colorectal cancer (CRC) is the third most common malignancy worldwide and one of the leading causes of cancer-related death [1].The liver is considered to be the most common site of colorectal cancer metastasis followed by the lung [2]. Because most of the mesentery drains into the hepatic portal vein system, with more than 50% of patients presenting with liver metastases. [3]. MSI is an important biomarker of colorectal cancer and has important diagnostic, prognostic, and predictive significance [4]. Microsatellites are single nucleotide or dinucleotide DNA repeat sequences. Instability is mainly characterized by single base pair insertions or deletions in these repeat sites, leading to extensive genomic instability [5]. Microsatellite states can be classified as high-frequency microsatellite instability, low-frequency microsatellite instability, and microsatellite stability. Fifteen percent of colorectal cancers are in microsatellite instability [6]. In colorectal cancer (CRC), MSI-H is associated with a better prognosis than MSI-L/MSS [7].

With the recent development of MSI detection technology and immunosuppressants in tumor therapy, researchers found that MSI-H tumors responded well to immunotherapy [8]. Immunotherapy has rapidly become the main treatment for various types of solid cancers [9], including rectal cancer (CRC) [10]. Programmed cell death-ligand 1 (PD-L1) Pemphumab and Navumab have been shown to be effective in patients with metastatic colorectal cancer with defective mismatch repair and high microsatellite instability [11]. There are two generally accepted methods for detecting microsatellite status. One is polymerase chain reaction detection, the other is immunohistochemical detection [12]. Molecular biology tests that rely on tissue samples are time-consuming and expensive, and tissue samples are often obtained through invasive endoscopic biopsies. This method has potential risks, such as bleeding [13]. Compared with biopsy, medical imaging can provide information about the overall heterogeneity of tumors and is a widely accepted alternative to biopsy. However, diagnostic images largely depend on the experience of radiologists. Therefore, it is necessary to develop non-invasive and cost-effective methods to predict MSI status.

Unlike traditional methods radiomics takes a non-invasive approach to predicting a patient's condition. Artificial intelligence is now widely used in medical image processing [14]. Radiomics can rapidly extract quantitative features from tomography image through high-throughput computing and then mine the correlation between these features and cancer diagnosis and prognosis [15].Imaging features can objectively and quantitatively characterize the tumor phenotype, which has potential predictive power for treatment outcomes and cancer genetics [16].

Radiomic features have been proven to be valuable as predictive indicators for the diagnosis, treatment response, and prognosis of various cancers, including CRC [17]. Many studies have proven that radiomics can noninvasively predict whether patients are in microsatellite instability. Aldo Rocca et al. helped physicians to predict the presence of liver metastases by analyzing CT radiological features [18]. Jennifer S. Golia Pernicka et al. performed radiomic analysis of patients' preoperative CT to determine whether the patients had MSI status [19]. Shuxuan Fan et al. used quantitative radiomic features extracted from CT to predict microsatellite status in Asian patients with stage II CRC [20]. Jingjun Wu et al. used dual-energy computed tomography imaging to perform a radiomics analysis on the images of iodine-based material decomposition to predict the MSI status of (CRC) [21].

The nomogram is a graphical representation of the mathematical model, which combines various important factors to predict the probability of clinical events [22]. It can be used as a reliable tool to predict clinical outcomes and adjuvant treatment decisions [23]. Yuan Hua et al. combined radiomics with clinical and pathological features to generate nomograms to predict the microsatellite instability status of patients [24]. Peng Yu et al. generated nomograms based on tumor and perineural CT combined with other information to evaluate MSI status in RC patients [25]. Qian Pei et al. constructed radiological nomograms for identifying preoperative microsatellite status in patients with combined CRC [13].

The above research distinguished only between MSI and MSS, or MSI-H and others. MSI-L cancers may overlap more with MSS than MSI-H cancers, but MSI-L still represents a distinct intermediate subgroup of colorectal cancers [24].

**Fig. 1:**
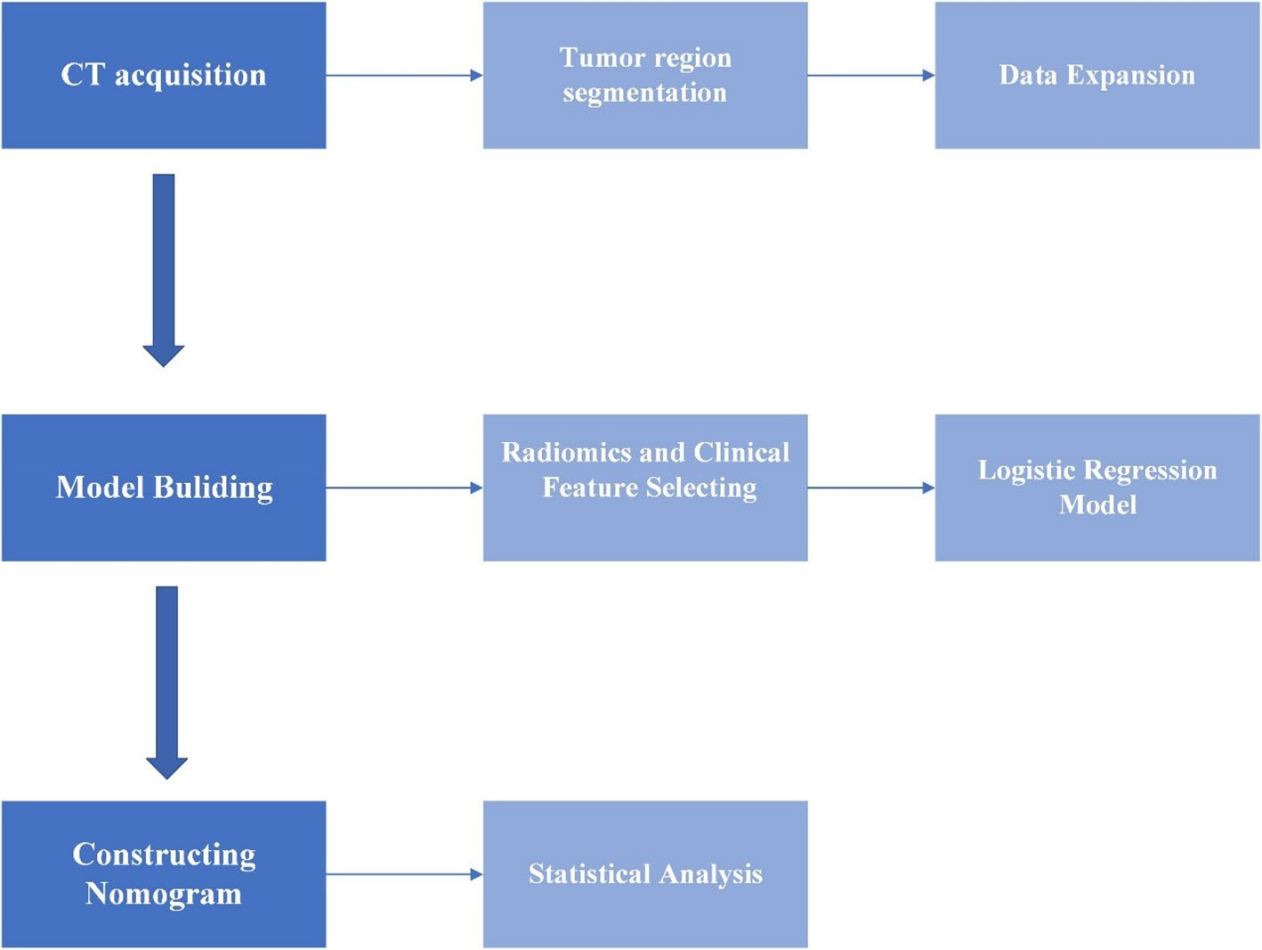
Flow chart

Therefore, it is also important to study whether the state of the microsatellite instability patient is high or low frequency. Although liver metastasis are identical to primary cancer in terms of genetic status (including MSI status), there are few reports studying MSI status in patients with liver metastasis from colorectal cancer based on CT images. To our knowledge, current studies of microsatellite status in patients with liver metastasis from colorectal cancer do not combine clinical features with radiomic features. In this study, clinical information was added to traditional radiomics to form a combined model. The experimental process is shown in Fig. 1. This study aimed to use enhanced CT images for preoperative noninvasive prediction of microsatellite status in patients with liver metastasis from colorectal cancer.

## Materials and methods

### Patients

The Institutional Review Board of Hebei University Hospital approved this retrospective study. All patients signed the informed consent form. All methods comply with relevant guidelines and regulations. Patients with CRLM lesions with typical liver metastasis confirmed by surgery, biopsy, or imaging (ultrasound, CT, or MRI) were included in the study. Determine the patient's microsatellite status by immunohistochemistry.

### CT image acquisition

The instruments used for CT scanning are the GE Discovery HD750 64-scanner and the GE Optima CT680 scanner. The parameters for CT scanning are: section thickness 5 mm, spacing 0.992, scanning field of view 350 mm × 350 mm, matrix 512 × 512, tube voltage 100–120 kV, tube current 160–300 mA. Contrast media were administered intravenously through the elbow at a flow rate of 3.0–3.5 mL/s. The dose was 0.5 mg per kg of body weight. The scanning times for the arterial phase, venous phase, and delayed phase were 30–35 s, 50–60 s, and 180 s, respectively, after the injection of contrast agent. The portal-venous phase of enhanced CT was selected for analysis.

### Region of interest (ROI) segmentation

Two radiologists (Doctors 1 and 2) used ITK-SNAP software to depict enhanced CT images of the portal venous phase. All doctors ignored patients' MSI status. The delineated area should contain as much of the tumor as possible but not surrounding normal or other tissue. Then generated a two-dimensional ROI.

### Data preprocessing

Because the scanning parameters of CT images were different, we preprocessed the images before the experiment. A total of 15 patients with MSI-H and 118 patients with MSI-L were included in the study. Due to the large difference between the two groups, the sample imbalance will result in a classification with a small sample size that contains too few features, making it difficult to extract patterns from them. Even if a classification model is obtained, it is prone to overfitting problems caused by overreliance on a limited data sample, and the classification model is heavily biased. Therefore, data expansion was used in this experiment, and the tumor section was enlarged or reduced to increase the MSI-H images to 26 cases, which improved the data imbalance.

### Statistical analysis

Standard deviation was used to represent measurement data and t-test was used to test. Count data is expressed as the number of cases (n) or percentage (%) and tested by chi-square. The consistency of imaging features within and between observers were evaluated using correlation coefficients (ICC) Doctors 1 and 2 randomly selected 30 CT images for ROI segmentation. ICC > 0.75 indicates that feature extraction has good consistency. The segmentation of the remaining images is done by doctor 1. *P* < 0.05 indicates that the difference is statistically significant. The model classification effectiveness of MSI-H and MSI-L CRLM was evaluated by receiver operator characteristic (ROC) curve analysis. The larger the area under the ROC curve, the higher the diagnostic efficiency. We calculated Area Under Curve (AUC), accuracy (ACC), specificity (SPE), and F1_score (F1).

### Model building

Radiomic features extraction was performed on the images and contour ROI files. Predictive models for identifying MSI-H and MSI-L CRLM were developed using a highly interpretable logistic regression classifier that is commonly used in medical classification.

We designed six prediction models using available preoperative data, and these models were built using a logistic regression classifier. The data were randomly divided into a training set and a test set; 70% of the data was used to design the prediction model, and the remaining 30% was used to evaluate the performance of the model.

### Construction of nomogram

The score of a variable in a nomogram depends on the contribution of that variable to the outcome. Then we can add the scores to get the total score. Finally, the prediction value of the individual is calculated through the functional transformation relationship between the total score and the probability of the results. For example, a positive biopsy, recurrence probability, or survival probability.

## Results

### Clinical Features

From Hebei University Hospital we collected CRLM CT image data for 104 patients, from January 2017 to December 2020. There were 15 patients with MSI-H CRLM and 118 patients with MSI-L CRLM. The clinicopathological features of the patient are shown Table 1.

**Table 1:** Clinical data of the training and validation groups

Variables	Training group(*n* = 101)	Validation group(*n* = 43)	P-value
**Gender** Female**/** Male	42(42%)/59(58%)	15(35%)/28(65%)	0.843
**Age**	58.43 ± 10.9	54.4 ± 10.7	0.038
**CEA**	60.01	73.8	> 0.999
**CA724**	34.47	21.74	0.564
**Tumor Location**			0.007
1	33	8	
2	5	6	
3	16	1	
4	17	5	
5	29	23	

*Abbreviations*: 1 of tumor location indicates Right colon; 2 of tumor location indicates Left colon;3 of tumor location indicates Partial sigmoid colon and rectum; 4 of tumor location indicates Sigmoid colon; 5 of tumor location indicates Rectum

### Feature selection

**Fig. 2:**
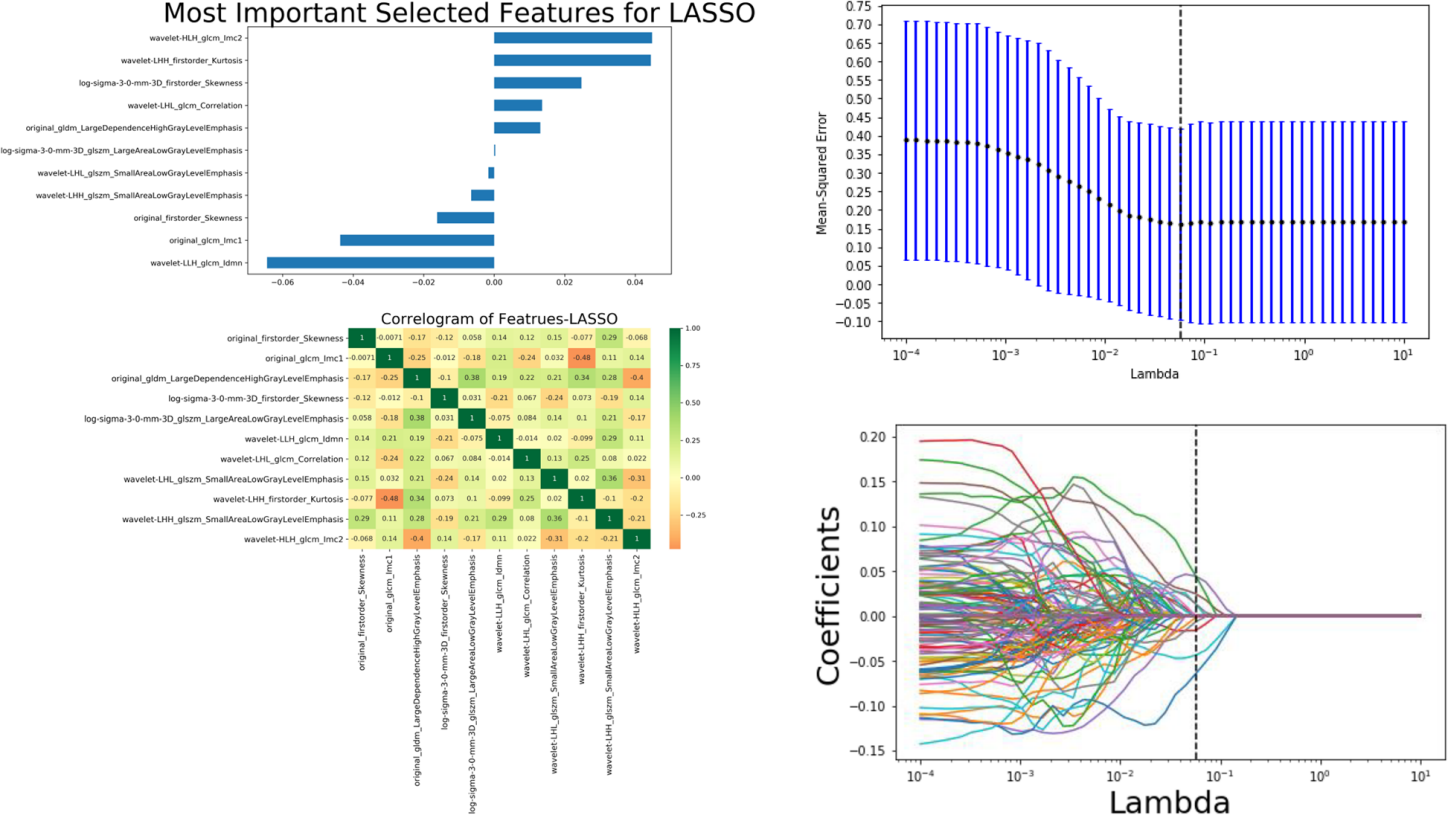
Feature selection in expanded data

A total of 967 features were extracted by radiomics, including shape features, first-order statistical features, second-order texture features, and wavelet features. We used the least absolute shrinkage and selection operator (LASSO) to select the best feature among all features. Figure 2 shows the feature heat map of Lasoo selected features, coefficient plot and cross-validation plot. A total of 13 potential predictors were selected in the original data group.

### Model validation

**Fig. 3:**
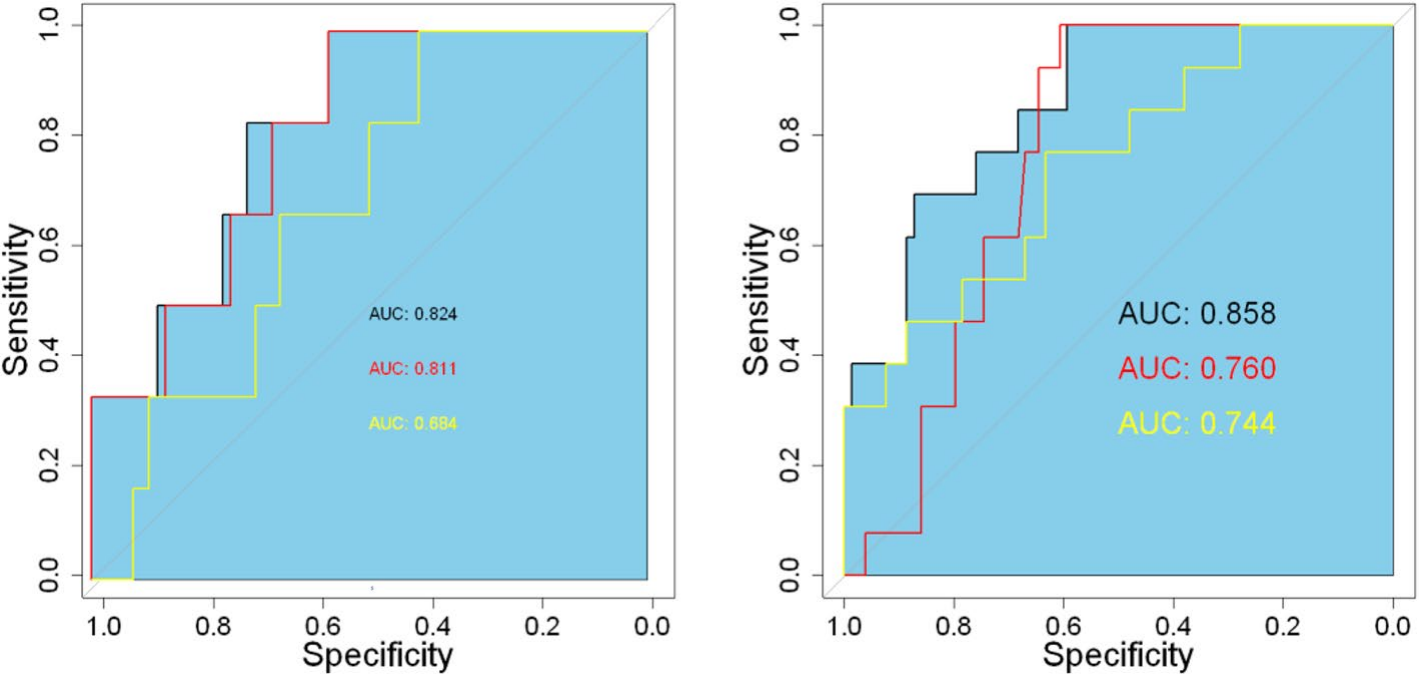
Receiver Operating Characteristic Curve (ROC). The (**A**) shows the ROC curves of three models in original data validation sets, and the (**B**) shows the ROC curves of three models after data expansion validation sets. The ROC curve for modeling combined with clinical and radiologic information is black; the ROC curve of the clinical information establishment model is red, and the ROC curve of the radiation group information modeling is yellow

We constructed a simple clinical model (OCM), a simple radionics model (ORM), and radiomic features confusing the clinical features model (OJM) from the original data. There are also three models in the expanded data: the simple clinical model (ECM), the simple radiomics model (ERM), and the radiomic features confusing clinical features model (EJM). The radiomics model includes radiomic features. The clinical logistic regression model includes clinical features. The clinical features included the primary tumor focus (left half rectum, right half rectum, part of the sigmoid colon and rectum, sigmoid colon and rectum) and age, with *P* < 0.05. The joint model includes radiomic features and clinical features. Figure 3 shows the receiver operating characteristic curve for models.

Model performance was compared between models using, specificity, and F1-score in validation group. The results are shown in Table 2.

**Table 2:** Clinical data of the training and validation groups

	**AUC**	**ACC**	**SPE**	**F1**
OCM	0.811	0.74	0.74	0.63
ORM	0.684	0.80	0.83	0.71
OJM	0.824	0.83	0.83	0.75
ECM	0.760	0.86	0.79	0.82
ERM	0.744	0.86	0.75	0.72
EJM	0.858	0.84	0.88	0.86

*Abbreviations*: OCM is a logistic regression model using the clinical information from the raw data; ORM is a logistic regression model using the radiomics information from the raw data; OJM is a logistic regression model combining the clinical and radiomics information from the raw data; ECM is a logistic regression model using the clinical information from the augmented data; ERM is a logistic regression model using the radiomics information from the augmented data; EJM is a logistic regression model combining the clinical and radiomics information from the augmented data. EJM is a logistic regression model that combines clinical and radiomic information from the augmented data

### Nomogram construction

**Fig. 4:**
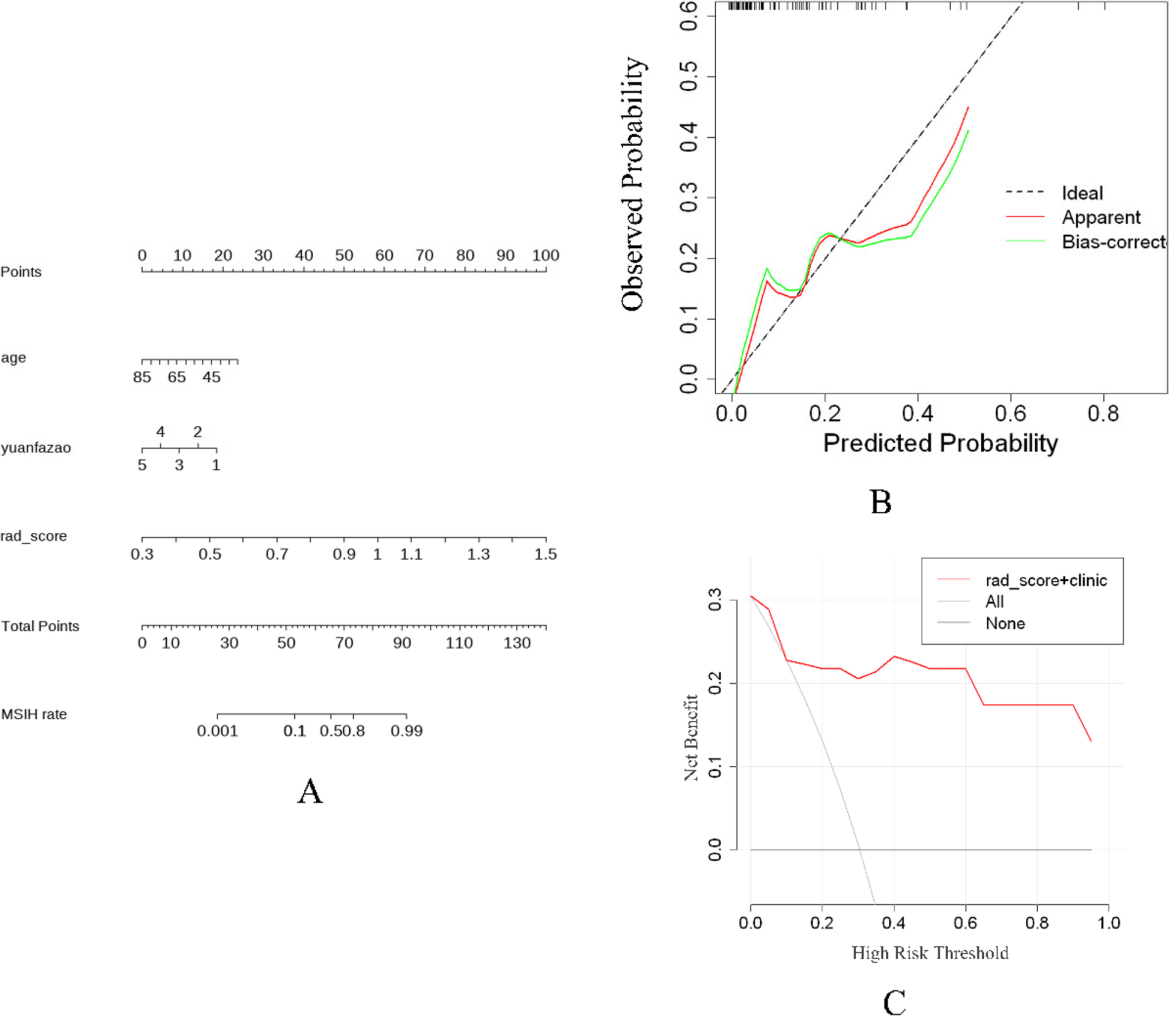
**A** was a nomogram. **B** was the calibration curve for the nomogram. The diagonal dashed reference line represents a perfect estimated MSI status for an ideal model. Solid lines represent the estimated MSI status of the nomogram. **C** was the decision curve analysis for the nomogram. The black line indicates the assumption that all patients are diagnosed not to be MSI-H, and the grey line indicates the assumption that all patients are diagnosed to be MSI-H. The red line represents the nomogram

We build a visual nomogram using rad_score and clinical features. Age and the tumor's primary site were selected. Rad_score were generated using the selected 11 features in the expanded data group. In the nomogram, the risk factors of each patient were located on a variable axis, and a vertical line is drawn up to determine the score of each risk factor. The total score is calculated by summing the scores of the selected variables, according to which the probability of high-frequency microsatellite instability in the corresponding patient can be calculated. The higher the score, the greater the probability that the patient with MSI-H. Figure 4 shows the Nomogram, the decision curve analysis and calibration curve.

## Discussion

We developed a radiomics prediction model using CT images of 104 patients with colorectal cancer liver metastasis from the Hebei University Hospital. Our finding suggests that this radiomics approach has the potential to assess MSI status. Radiomic features obtained from CRLM CT images. The model, which combines the radiomic features and clinical features, can effectively distinguish colorectal cancer patients with liver metastasis in MSI-H CRLM and MSI-L CRLM. Our results show that the prediction performance of the EJM is better. A user-friendly nomogram was constructed based on this joint model to provide effective guidance for the clinical treatment of CRLM patients with unknown MSI status.

For patients with liver metastases from colon cancer, early radical surgical resection is an important prerequisite to improve the five-year survival rate. With the development of medical technology various treatment methods have been improved, and for patients with simultaneous metastases the application of minimally invasive laparoscopic surgery can perform minimally invasive resection of both the primary and liver metastases [26].Robot-assisted surgery enables precise cutting, separation and suturing [27], Minimally invasive robotic-assisted colorectal and liver resection has also been shown to be feasible and safe [28].

MSI-H CRLM have a good prognosis and will not benefit from adjuvant chemotherapy based on 5-FU [29].Studies have shown that MSI-H CRC have significant, stage-independent, multifactor survival advantages. Colorectal cancer patients with MSI-H can benefit from immunotherapy because of their increased immune infiltration and the higher load of new antigens [30]. Therefore, the state of microsatellite instability in patients with colorectal cancer liver metastasis before treatment is important to the patient's prognosis.

Nomogram transforms complex regression equations into visual graphs to make the results of predictive models more readable and facilitate the evaluation of patients. Because the nomogram is intuitive and easy to understand, it has gradually been adopted and applied in medical research and clinical practice. Qian Pei et al. developed a nomogram based on CT images to predict the microsatellite status of colorectal cancer patients before surgery [13]. Harini Veeraraghavan et al. identified endometrial carcinoma with a DNA mismatch repair defect or high tumor mutation load through radiomic features based on contrast-enhanced computed tomography [31]. Liang Xiuqun et al. constructed a model based on computed tomography images and clinical features for preoperative prediction of MSI patients [32]. Liang Xiuqun et al. constructed a model based on computed tomography images and clinical features for preoperative prediction of MSI patients [33]. Huang Z-Xing et al. developed a radiomics model based on T2WI images for preoperative diagnosis of MSI patients [6]. Although the biological characteristics and pathological types of metastatic tumors are consistent with those of primary tumors, there are few reports on predicting MSI in colorectal cancer patients with liver metastasis based on CT images. Therefore, we use a joint model based on CT images for the non-invasive detection of microsatellite instability in patients with CLRM. Our research shows that the joint model performs well in distinguishing MSI-H and MSI-L states. The limitation of this study is that it is a retrospective single-center study with a small sample size. Although the study is flawed, it combined the real data of patients with colorectal cancer liver metastases in clinical practice to guide the treatment and prognosis of patients.

## Conclusion

We investigated a joint model that could non-invasively predict microsatellite instability in CRLM. The model could assist in clinical decision-making by providing personalized treatment plans for patients with liver metastasis from colorectal cancer.

## Data Availability

The datasets analyzed during the current study are not publicly available due to its private nature but are available from the corresponding author on reasonable request.
